# Identifying RBM47, HCK, CD53, TYROBP, and HAVCR2 as Hub Genes in Advanced Atherosclerotic Plaques by Network-Based Analysis and Validation

**DOI:** 10.3389/fgene.2020.602908

**Published:** 2021-01-15

**Authors:** Chiyu Liu, Haifeng Zhang, Yangxin Chen, Shaohua Wang, Zhiteng Chen, Zhaoyu Liu, Jingfeng Wang

**Affiliations:** ^1^Department of Cardiology, Sun Yat-sen Memorial Hospital, Sun Yat-sen University, Guangzhou, China; ^2^Laboratory of Cardiac Electrophysiology and Arrhythmia in Guangdong Province, Guangzhou, China; ^3^Guangdong Provincial Key Laboratory of Malignant Tumor Epigenetics and Gene Regulation, Sun Yat-sen Memorial Hospital, Sun Yat-sen University, Guangzhou, China

**Keywords:** atherosclerosis, bioinformatics, weighted gene co-expression network analysis, differentially expressed genes, gene ontology (GO), Kyoto Encyclopedia of Genes and Genomes

## Abstract

**Background:** Atherosclerotic cardiovascular diseases accounted for a quarter of global deaths. Most of these fatal diseases like coronary atherosclerotic disease (CAD) and stroke occur in the advanced stage of atherosclerosis, during which candidate therapeutic targets have not been fully established. This study aims to identify hub genes and possible regulatory targets involved in treatment of advanced atherosclerotic plaques.

**Material/Methods:** Microarray dataset GSE43292 and GSE28829, both containing advanced atherosclerotic plaques group and early lesions group, were obtained from the Gene Expression Omnibus database. Weighted gene co-expression network analysis (WGCNA) was conducted to identify advanced plaque-related modules. Module conservation analysis was applied to assess the similarity of advanced plaque-related modules between GSE43292 and GSE28829. Gene Ontology (GO) and Kyoto Encyclopedia of Genes and Genomes (KEGG) enrichment analysis of these modules were performed by Metascape. Differentially expressed genes (DEGs) were mapped into advanced plaque-related modules and module membership values of DEGs in each module were calculated to identify hub genes. Hub genes were further validated for expression in atherosclerotic samples, for distinguishing capacity of CAD and for potential functions in advanced atherosclerosis.

**Results:** The lightgreen module (MElightgreen) in GSE43292 and the brown module (MEbrown) in GSE28829 were identified as advanced plaque-related modules. Conservation analysis of these two modules showed high similarity. GO and KEGG enrichment analysis revealed that genes in both MElightgreen and MEbrown were enriched in immune cell activation, secretory granules, cytokine activity, and immunoinflammatory signaling. RBM47, HCK, CD53, TYROBP, and HAVCR2 were identified as common hub genes, which were validated to be upregulated in advanced atherosclerotic plaques, to well distinguish CAD patients from non-CAD people and to regulate immune cell function-related mechanisms in advanced atherosclerosis.

**Conclusions:** We have identified RBM47, HCK, CD53, TYROBP, and HAVCR2 as immune-responsive hub genes related to advanced plaques, which may provide potential intervention targets to treat advanced atherosclerotic plaques.

## Introduction

Atherosclerosis (AS) is the most common underlying cause of cardiovascular diseases, and accounted for a quarter of global deaths (Lee et al., 2019). Clinically the most significant and dangerous process in atherosclerotic cardiovascular disease like coronary atherosclerotic disease (CAD) and stroke is the growth and development of advanced atherosclerotic lesions, which causes either arterial lumen stenosis and blood flow obstruction or plaque rupture to enhance atherosclerotic thromboembolism (Fuster et al., 2005; Kovanen, 2019). However, no effective drugs have yet been available to reverse advanced plaques formation (Gaurav et al., 2015).

High throughput sequencing technology for detecting gene expression is an effective tool to reveal the underlying genes and biological processes during atherosclerotic plaque formation (Tan et al., 2017). Recently, several atherosclerotic gene expression profiling studies have been performed. Chen et al. (2020) identified THRAP3 and RBM43 as potential diagnostic and prognostic targets for ischemic event occurrence in carotid atherosclerosis. Jiao et al. (2020) identified CD40, F11R, TNRC18, and CAMK2G as surrogate diagnostic biomarkers for CAD in non-diabetic patients. Zhang et al. (2019) have identified TNPO1, RAP1B, ZDHHC17, and PPM1B as targets of CAD-related miRNAs. These studies were based on analysis of AS-related expression profile data in human peripheral blood, which were inclined to screen biomarkers for diagnosis and prognosis. However, gene expression profile analysis in diseased tissue is more helpful to reveal the mechanism of disease progression. Presently, there are only a few tissue-based studies of advanced plaque-related expression profile analysis. These studies focused on protein-protein interaction network (PPI) or transcription factor co-regulation network, both of which were based on previous experimental evidence (prior knowledge) (Lin et al., 2014; Wang et al., 2014; Tan et al., 2017; Liu et al., 2018). However, these analysis neglects gene expression information and clinical traits of samples under specific disease condition (Feltrin et al., 2019). Weighted gene co-expression network analysis (WGCNA) is an advanced bioinformatics analysis method that relies exclusively on the expression data values, making it possible to find all possible gene expression correlation under specific disease condition (Zhang and Horvath, 2005; Feltrin et al., 2019). Besides, WGCNA makes full use of the phenotypic traits of samples to identify trait-related gene sets, which may further improve the biological and clinical significance beyond classical methods (Miao et al., 2018; Shi et al., 2019).

In the present study, two human advanced atherosclerotic plaque-related expression profile datasets were obtained. Then advanced plaque-related co-expressed gene modules were identified, whose underlying biological processes were revealed. Finally, the hub genes in advanced plaques were identified and validated for expression in atherosclerotic samples, for distinguishing capacity of coronary atherosclerotic disease and for potential functions in advanced atherosclerosis, which provides new insight for AS progression and promising intervention targets.

## Materials and Methods

### Data Analysis Workflow

**Figure 1:**
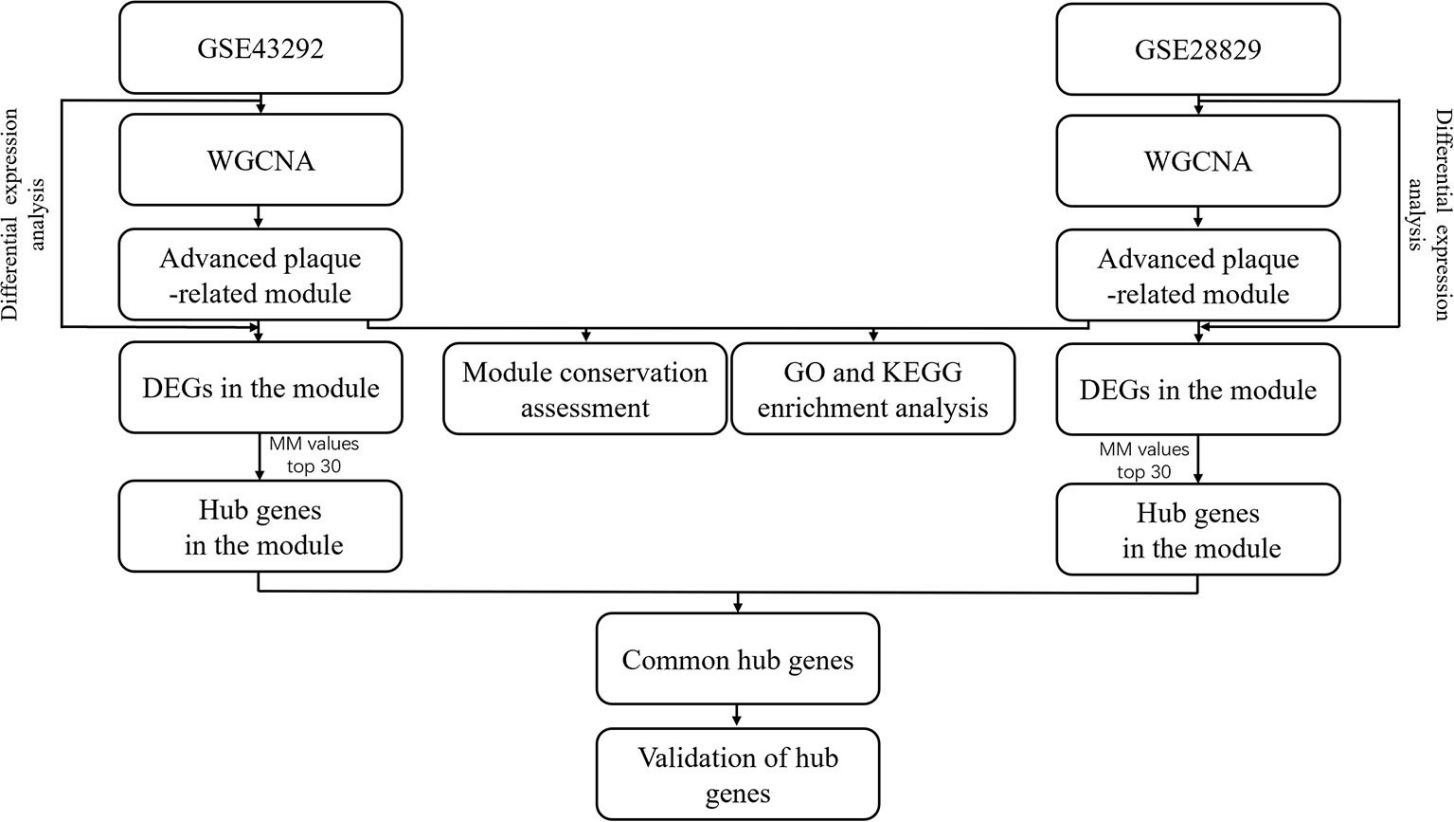
Flowchart of data analysis.

The flowchart of data analysis was presented in Figure 1. After advanced atherosclerosis-related datasets, GSE43292 and GSE28829, were obtained, WGCNA was applied to identify their own advanced plaque-related module. Module conservation analysis was applied to assess the similarity of advanced plaque-related modules between GSE43292 and GSE28829. Meanwhile, GO and KEGG enrichment analysis was applied to reveal the underlying biological functions of these modules. After differential expression analysis of each dataset, the identified DEGs were mapped into advanced plaque-related modules and module membership values of DEGs in each module were calculated to identify hub genes. Hub genes were further validated for expression level in advanced atherosclerotic tissues by PCR and differential expression analysis, for distinguishing capacity of coronary atherosclerotic disease by ROC analysis and for potential biological functions by Gene Set Enrichment Analysis.

### Data Sources

The datasets of GSE43292, GSE28829, GSE18443, GSE31947, and GSE12288 were obtained from the Gene Expression Omnibus (GEO) database (http://www.ncbi.nlm.nih.gov/geo/). In dataset GSE43292, samples collected from carotid endarterectomy in hypertensive patients were divided into early lesions group (stages I and II of the Stary classification) and advanced plaques group (stage IV and over of the Stary classification). This dataset was performed using the platform GPL6244 [Affymetrix Human Gene 1.0 ST Array, transcript (gene) version]. In dataset GSE28829, atherosclerotic carotid artery segments obtained during autopsy were also divided into early lesions group (stages I and II) and advanced plaques group (stage IV or above). This dataset was performed using the platform GPL570 [Affymetrix Human Genome U133 Plus 2.0 Array]. Both dataset GSE18443 and GSE31947 contained aortic samples from ApoE^−/−^ mice fed with high fat diet for 16 weeks (advanced plaques group) and wildtype mice fed with high fat diet for 16 weeks (control group). These samples were sequenced using the platform GPL3677 (Rosetta/Merck Mouse 44k 1.0 microarray). Dataset GSE12288 contained peripheral blood samples from patients with coronary atherosclerotic disease (CAD group, coronary artery stenosis >50% in at least one major coronary artery) and controls (Non-CAD group, no angiographically detectable coronary artery stenosis). This dataset was performed using the platform GPL96 [Affymetrix Human Genome U133A Array]. GSE18443, GSE31947, and GSE12288 were used as validation datasets.

### Data Preprocessing

Data files stored in a raw format (.CEL files) were preprocessed using Robust Multichip Average algorithm (RMA) of oligo package within Bioconductor (http://www.bioconductor.org/) in *R3.5.2* software (Carvalho and Irizarry, 2010; Wang et al., 2014). After background adjustment, normalization, and log transformation by RMA, the raw data was converted into a gene expression matrix. The “nsFilter” function contained in genefilter package was applied to filter out 50% probes based on IQR. Then the probe IDs were replaced by gene symbols. If more than one probe corresponded to one gene, the median expression value of all probes was analyzed.

### Constructing Co-expressed Gene Modules and Identifying Advanced Plaque-Related Modules

The analysis processes of WGCNA included: (1) Samples were clustered by hierarchical clustering analysis and samples not conforming to the experimental grouping (outliers) were detected. (2) Appropriate soft threshold value (β) was chosen to make the co-expression network approximate biologically significant scale-free topology (scale independence >0.8). (3) The co-expression similarity between gene i and j was defined as adjacency value between genes: a_ij_ = |S_ij_|^β^ (S_ij_ = |cor(i, j)|). (4) The adjacency matrix was transformed into a topological overlap matrix (TOM), and modules were detected by hierarchical clustering analysis of the gene dendrogram. (5) The module eigengene (ME) value of each module was summarized by the first principal component of the module expression levels. The advanced plaque-related modules were identified by correlating ME values with the clinical traits of samples using hierarchical clustering analysis and spearman correlation analysis. Advanced plaque-related module was defined as the module showing the highest correlation coefficient with advanced plaque (*P* < 0.05) and the closest location with advanced plaque on the hierarchical cluster dendrogram.

### Assessing the Similarity of Advanced Plaque-Related Modules Between Dataset GSE43292 and GSE28829

The module conservation analysis processes included: (1) Common soft threshold value (β) was chosen to make the co-expression network of both datasets approximate biologically significant scale-free topology. (2) Common soft threshold value was used to calculate adjacency values in the individual datasets. (3) The adjacency matrix in the individual datasets was transformed into topological overlap matrix (TOM). (4) The TOM in GSE28829 was scaled such that the 95th percentile equals the 95th percentile of the TOM in GSE43292. (5) The consensus TOM was calculated by taking the component-wise (“parallel”) minimum of the TOMs in individual datasets. (6) Based on consensus TOM, consensus modules were detected by hierarchical clustering analysis of the gene dendrogram. (7) The overlapping gene counts of each pair of GSE43292 (or GSE28829)—consensus modules were calculated to relate the modules in GSE43292 (or GSE28829) to the consensus modules, and the Fisher's exact test was used to assign a *p*-value to each of the pairwise overlaps. (8) If a module in dataset GSE43292 had the same consensus counterpart with a module in dataset GSE28829, these two modules were considered to be similar.

### Functional Enrichment Analysis of Genes in Advanced Plaque-Related Modules

To reveal the underlying biological functions of the advanced plaque-related modules, genes in them were introduced into the DAVID database (https://david.ncifcrf.gov/) for Gene Ontology (GO) enrichment analysis and Metascape database (https://metascape.org) for Kyoto Encyclopedia of Genes and Genomes (KEGG) enrichment analysis (Zhou et al., 2019). GO describes the biological processes (BP), subcellular localization (CC), and molecular function (MF) enriched in gene sets (Ashburner et al., 2000; Mi et al., 2017; Consortium, 2019). And KEGG describes the pathways enriched in gene sets (Kanehisa and Sato, 2020). Terms with a *P* < 0.01, a minimum overlap of 3 and a minimum enrichment of 1.5 were identified.

### Identifying Hub Genes in Advanced Plaques

Differentially expressed genes (DEGs) of GSE43292 and GSE28829 were identified based on |log_2_fold change (FC)| > 1 and *P* < 0.05 by using Limma package (Smyth, 2005). DEGs were then intersected with genes of advanced plaque-related module in each dataset to obtain DEGs in advanced plaque-related modules. Module membership (MM) value of each gene was calculated by WGCNA and the genes with top 30 MM values were identified as hub genes in each module.

### Aortic Tissue Collection of Animal Models

After being fed with high fat diet or normal chow for 16 weeks, ApoE^−/−^ mice were fasted for 14 h, and then anesthetized and euthanized. The aortic tissues were removed from the ascending aorta to the ileal bifurcation and snap frozen in liquid nitrogen for RNA analysis.

### RNA Extraction and Quantitative Real-Time PCR

Total RNA from aortic tissues was extracted using TRIzol reagent (Invitrogen) and purified using isopropanol, 75% ethanol and RNase-free water following the introductions of manufacturer. cDNA was synthesized using the PrimeScript™ RT Master Mix kit (Takara). Quantitative real-time PCR was performed using the TB Green® Premix Ex Taq™ kit (Takara) by Applied Biosystems Quanstudio DX (Thermo Fisher Scientific) after setting up the appropriate protocol. β-actin was used as an internal reference. The 2^−ΔΔCt^ method was applied to analyze the results of PCR. All the primers were designed online (https://pga.mgh.harvard.edu/primerbank/) and were listed in Supplementary Table 1.

### Differential Expression Validation of Hub Genes in Dataset GSE18443, GSE31947, and GSE12288

Differential expression analysis of hub genes was performed by limma package in *R3.5.2* software. *P* < 0.05 was used to screen differentially expressed hub genes.

### Receiver Operating Characteristic (ROC) Curve Validation of Hub Genes in Dataset GSE12288

ROC was plotted and area under the curve (AUC) was calculated by *GraphPad Prism 8* software to evaluate the capability of hub genes to distinguish samples of CAD group from those of non-CAD group. Efficacy evaluation: AUC = 0.5, non-efficiency; AUC >0.5 but <0.7, modest-efficiency; AUC >0.7, high-efficiency.

### Gene Set Enrichment Analysis (GSEA) of Hub Genes

GSEA run in the *GSEA* software using dataset GSE28829. Based on the median expression of each hub gene, samples were divided into high expression group and low expression group. In run program settings, gene set “c2.cp.kegg.v6.2.symbols.gmt” was chosen as the reference gene set and the parameter “number of permutations” was set as 1,000. The top three gene sets with the lowest NOM (*p* < 0.01) were considered significantly enriched.

### Constructing Transcriptional Regulatory Network and Identifying Key Transcription Factors in Advanced Plaque-Related Modules

TRRUST v2 (www.grnpedia.org/trrust) is a database of reference TF–target regulatory interactions in humans and mice based on literature curation, which applies a network-based algorithm to prioritize key TFs for the given transcriptionally responsive genes (Han et al., 2018). The DEGs in advanced plaque-related modules were introduced into TRRUST v2 database and *P* < 0.001 was used to screen significantly enriched transcriptional regulatory networks.

### Constructing Protein-Protein Interaction (PPI) Network in Advanced Plaque-Related Modules

The DEGs in advanced plaque-related modules were introduced into STRING database (https://string-db.org/) to construct PPI network. The *Cytohubba* plugin of *Cytoscape* software was used to perform network analysis and identify top 10 genes with the highest degree.

## Results

### Construction of Co-expressed Gene Modules

**Figure 2:**
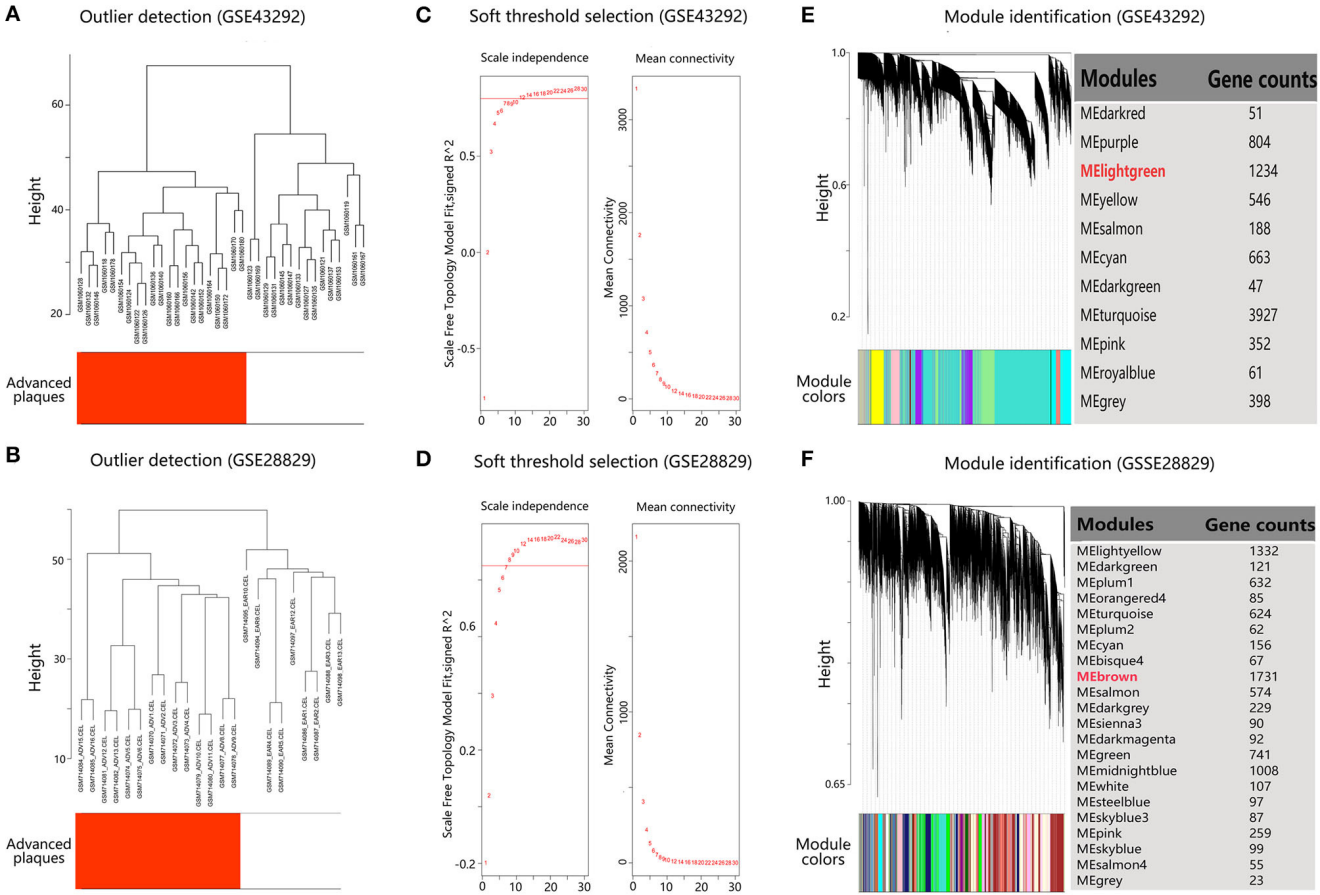
Construction of co-expressed gene modules in dataset GSE43292 and GSE28829. **(A,B)** Clustering dendrogram of samples to detect outliers. **(C,D)** Analysis of network topology for various soft threshold powers. The left panel shows the scale-free fit index (scale independence, y-axis) as a function of the soft threshold power (x-axis). The right panel displays the mean connectivity (degree, y-axis) as a function of the soft threshold power (x-axis). **(E,F)** Gene dendrogram obtained by average linkage hierarchical clustering. The color row underneath the dendrogram shows the module assignment determined by the Dynamic Tree Cut.

To identify co-expressed gene sets, WGCNA was applied. The microarray quality was first evaluated by sample clustering. As shown in Figures 2A,B, samples were clustered into two clusters in each dataset, corresponding to the early lesions group and advanced plaques group, respectively. No outliers were detected in the clusters. Next, 12 and 8 were chosen as the soft threshold for GSE43292 and GSE28829, respectively, which ensured a scale-free network (Figures 2C,D). As a result, 11 co-expressed modules in GSE43292 were identified and 22 co-expressed modules in GSE28829 were identified (Figures 2E,F).

### Identification of Advanced Plaque-Related Modules

**Figure 3:**
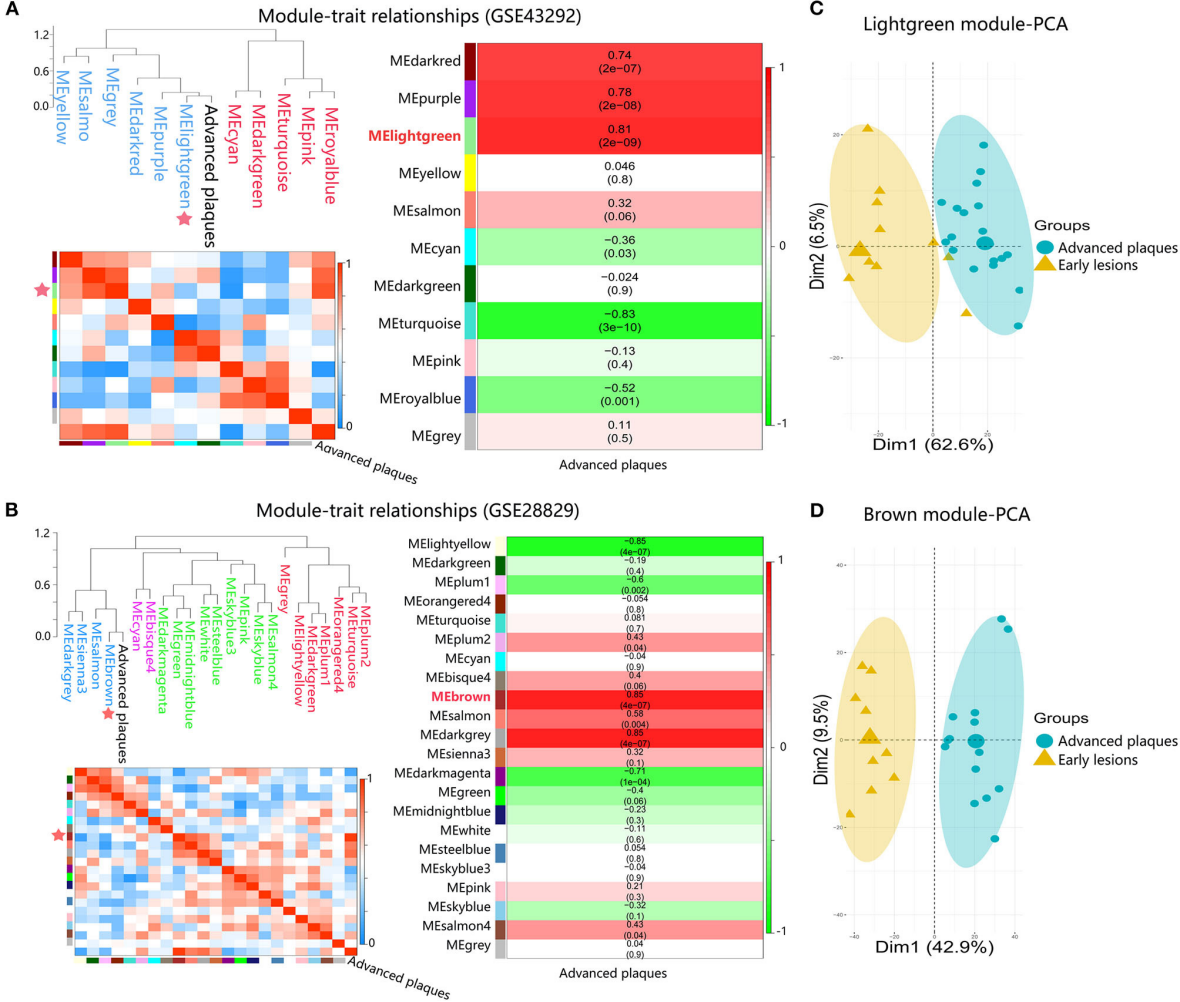
Identification of advanced plaque-related modules in dataset GSE43292 and GSE28829. **(A,B)** Module-trait associations. Branches of the dendrogram group together module eigengene values that are positively correlated. Each row and column in the lower left heatmap correspond to one module eigengene (labeled by color) or advanced plaques, in which blue color represents negative correlation, while red represents positive correlation. Each row in the right heatmap corresponds to a module eigengene, column to the advanced plaques. Each cell contains the corresponding correlation and *p*-value. The table is color-coded by correlation according to the color legend. **(C,D)** The PCA for genes within advanced plaque-related modules in response to advanced plaques.

To identify advanced plaque-related modules, module eigengene (ME) values of modules were correlated with the clinical traits of samples by applying average linkage hierarchical clustering algorithm and spearman correlation coefficient. As shown in Figures 3A,B, the lightgreen module (MElightgreen) in GSE43292 and the brown module (MEbrown) in GSE28829 showed the highest association with advanced plaques. Principal component analysis results showed that genes in MElightgreen and MEbrown could well distinguish samples of advanced plaques group from that of early lesions group, indicating that genes in these two modules contributed to plaque progression from early to advanced stages (Figures 3C,D). Hence, MElightgreen and MEbrown were identified as advanced plaque-related modules for GSE43292 and GSE28829, respectively.

### Module Conservation Analysis of MElightgreen and MEbrown

**Figure 4:**
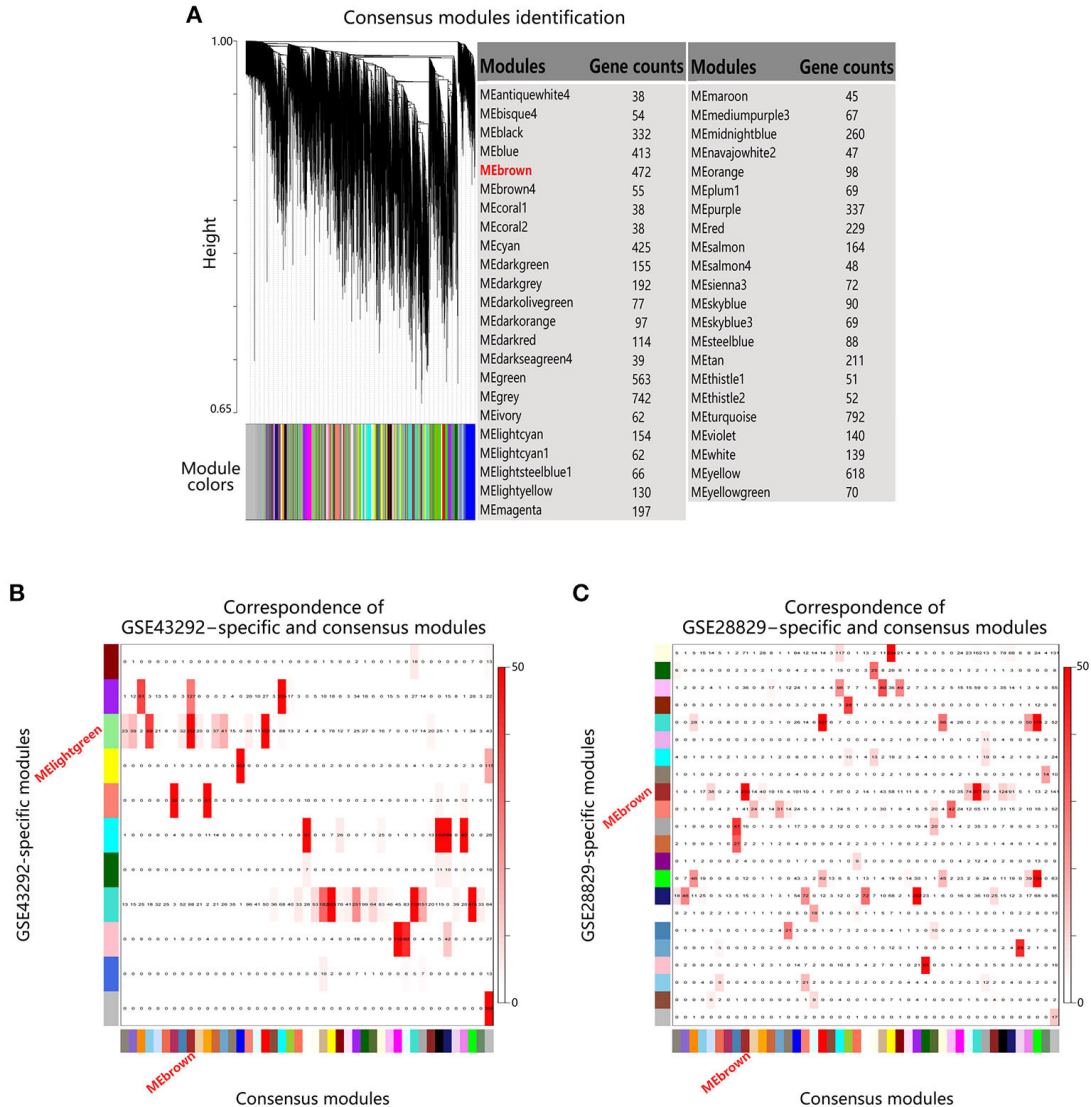
Module conservation analysis of MElightgreen and MEbrown. **(A)** Gene dendrogram obtained by hierarchical clustering based on consensus Topological Overlap. The color rows show the module assignments. **(B,C)** Correspondence of individual dataset-specific modules and the consensus modules. Each row of the table corresponds to one individual dataset-specific module (labeled by color as well as text), and each column corresponds to one consensus module. Numbers in the table indicate gene counts in the intersection of the corresponding modules. Coloring of the table encodes – log(p), with p being the Fisher's exact test *p*-value for the overlap of the two modules. The stronger the red color, the more significant the overlap is.

Next module conservation analysis was performed to validate the similarity between MElightgreen and MEbrown, which were derived from different datasets. As shown in Supplementary Figure 1, a power of 12 was chose as common soft threshold value to ensure scale-free topology for each dataset. And after scaling topological overlap matrixs (TOMs) of the two datasets for mitigating the effect of different statistical properties, they were comparable across datasets (Supplementary Figure 2). Subsequently, consensus TOM was calculated by combining the TOMs of two datasets and 45 consensus modules were identified (Figure 4A). By further calculating the overlapping gene counts between individual module in each dataset and consensus modules, MElightgreen in GSE43292 and MEbrown in GSE28829 had the most overlapping gene counts with the same module in consensus modules (Figures 4B,C), suggesting that MElightgreen in dataset GSE43292 and MEbrown in GSE28829 had high similarity.

### Functional Enrichment Analysis of Genes in Advanced Plaque-Related Modules

**Figure 5:**
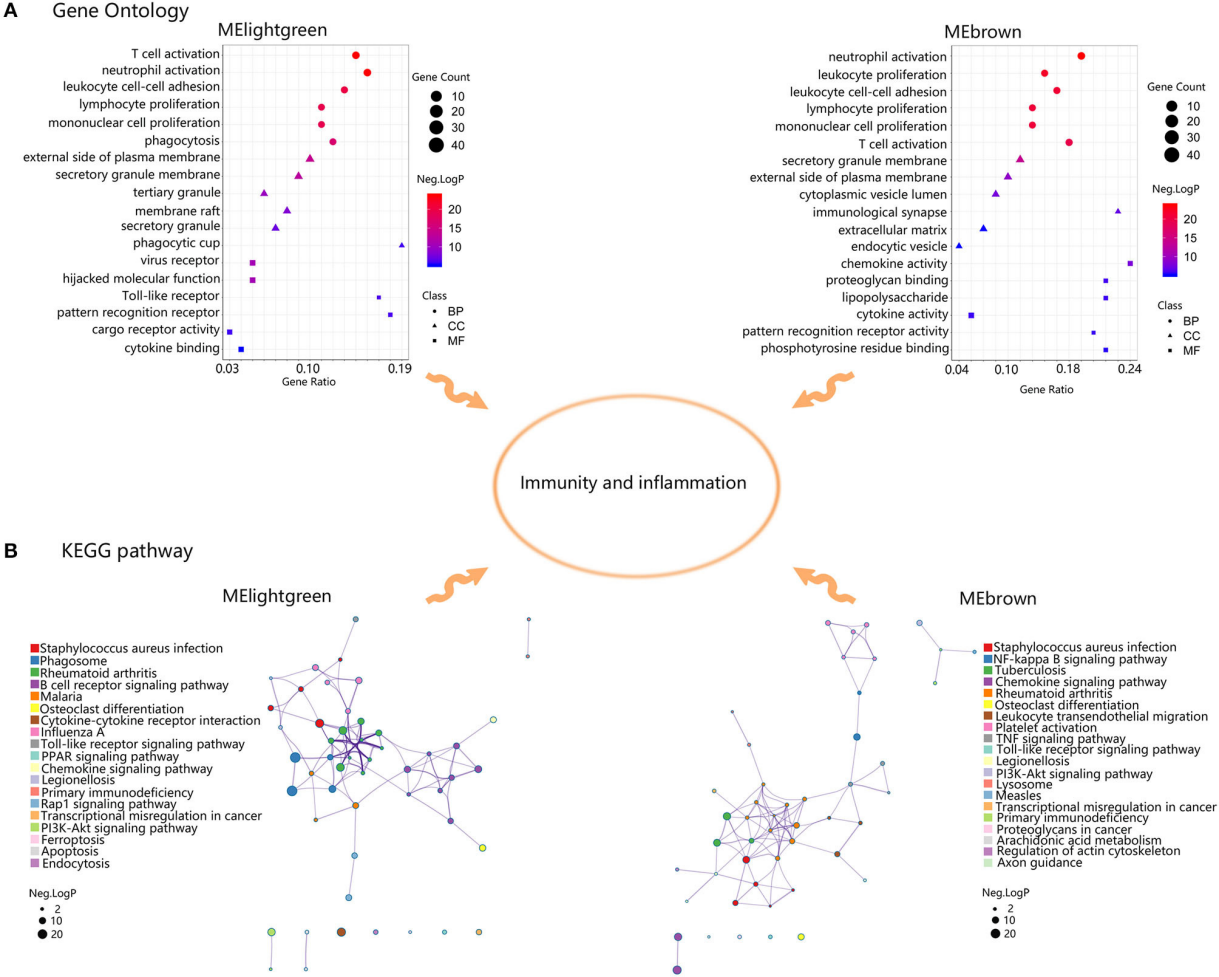
Functional enrichment analysis of genes in MElightgreen and MEbrown. **(A)** Gene Ontology enrichment results of MElightgreen and MEbrown. The sizes of the dots represent the counts of enriched module genes, and the dot color represents the negative Log10 (*p*-value). **(B)** Kyoto Encyclopedia of Genes and Genomes (KEGG) enrichment results of MElightgreen and MEbrown. The sizes of the dots represent the negative Log10 (*p*-value).

Then GO and KEGG analysis was executed to assess the functions of genes in MElightgreen and MEbrown (Figure 5). In MElightgreen, the GO terms of BP were enriched in immune cell activation, adhesion, proliferation, and phagocytosis. The GO terms of CC were enriched in plasma membrane, secretory granule, and phagocytic cup. The GO terms of MF were enriched in virus recepter, hijacked molecular function, Toll-like receptor, pattern recognition receptor, cargo receptor activity, and cytokine binding. The pathway terms were mainly enriched in immune response against infection, phagosome, rheumatoid arthritis, osteoblast differentiation, cytokine-cytokine receptor interaction, primary immunodeficiency, transcriptional misregulation in cancer, ferroptosis, apoptosis, endocytosis, and many signaling pathways like chemokine signaling pathway. In MEbrown, the GO terms of BP were enriched in immune cell activation, adhesion, proliferation. The GO terms of CC were enriched in plasma membrane, secretory granule, immunological synapse, extracellular matrix, and endocytic vesicle. The GO terms of MF were enriched in chemokine activity, proteoglycan binding, lipopolysaccharide, cytokine activity, pattern recognition receptor activity, and phosphotyrosine residue binding. The pathway terms were mainly enriched in immune response against infection, rheumatoid arthritis, osteoblast differentiation, leukocyte transendothelial migration, platelet activity, lysosome, transcriptional misregulation in cancer, primary immunodeficiency, proteoglycans in cancer, arachidonic acid metabolism, regulation of actin cytoskeleton, axon guidance, and many signaling pathways like chemokine signaling pathway. Collectively, these results suggested that genes within advanced plaque-related modules were involved in immune-related functions.

### Identification of Hub Genes in Advanced Plaque Progression

**Figure 6:**
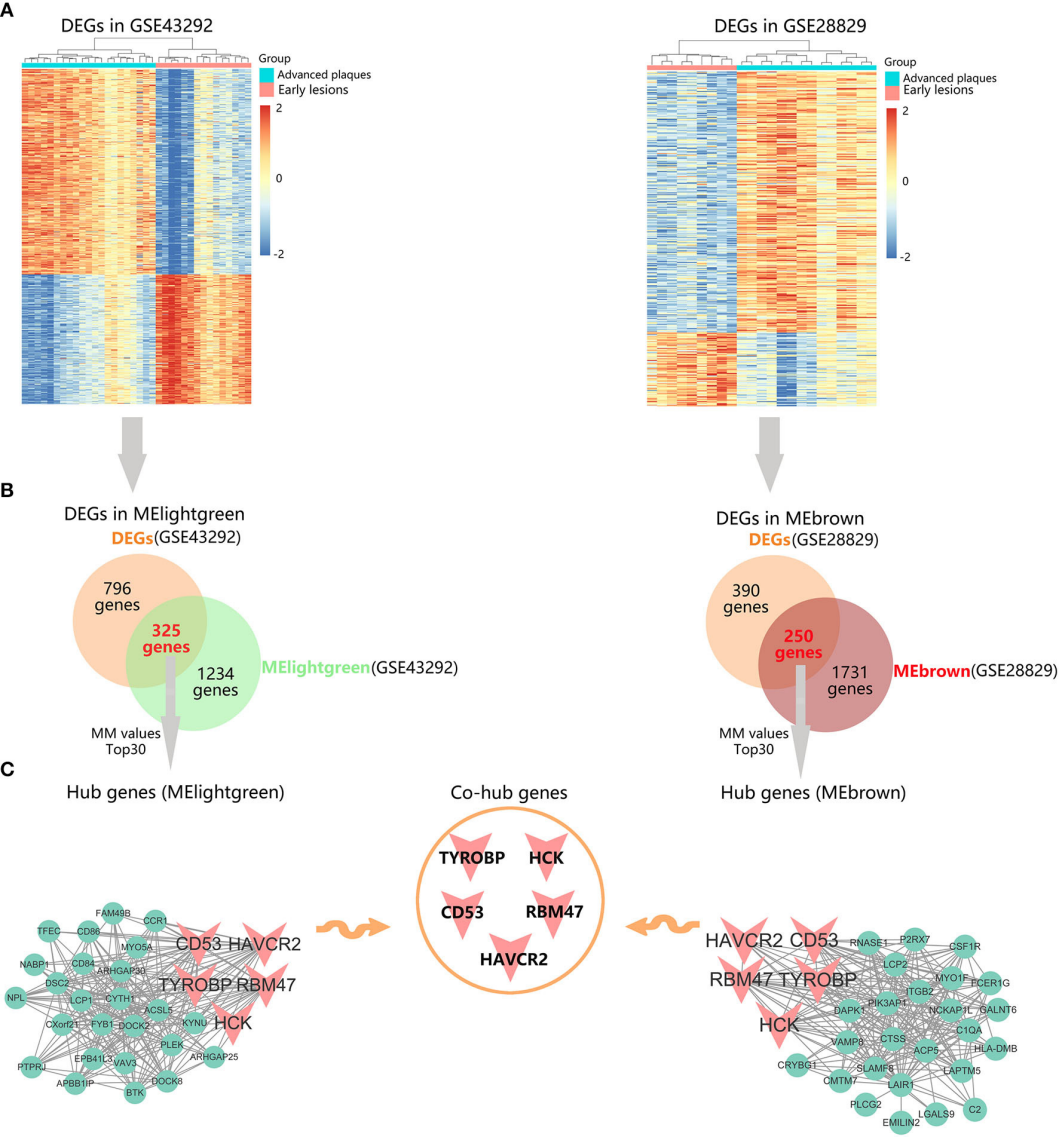
Identification of hub genes in advanced plaques. **(A)** Heatmaps of the differentially expressed genes (DEGs) in GSE43292 and GSE28829. **(B)** Venn diagrams of DEGs and advanced plaque-related module genes in GSE43292 and GSE28829. **(C)** Identification of intramodular hub genes whose module membership (MM) values rank top 30 among DEGs in corresponding modules.

To further identify hub genes in advanced plaque progression, we first mapped differentially expressed genes (DEGs) into advanced plaque-related modules. As shown in Figure 6A, with the threshold of |log_2_fold change (FC)| > 1 and *P* < 0.05, 796 DEGs were identified in GSE43292 with 483 upregulated and 313 downregulated. 390 DEGs were identified in GSE28829 with 304 upregulated and 86 downregulated. After intersection of these DEGs with advanced plaque-related modules, 325 DEGs were identified in MElightgreen and 250 DEGs were identified in MEbrown (Figure 6B). Further, the top 30 genes in MM values of MElightgreen and MEbrown were identified as their respective hub genes (Figure 6C and Supplementary Table 2). Notably, TYROBP, HCK, CD53, RBM47, and HAVCR2 were identified as common hub genes in the two datasets, indicating their key roles in advanced plaque formation.

### Validation of Hub Gene Expression in Advanced Atherosclerosis

**Figure 7:**
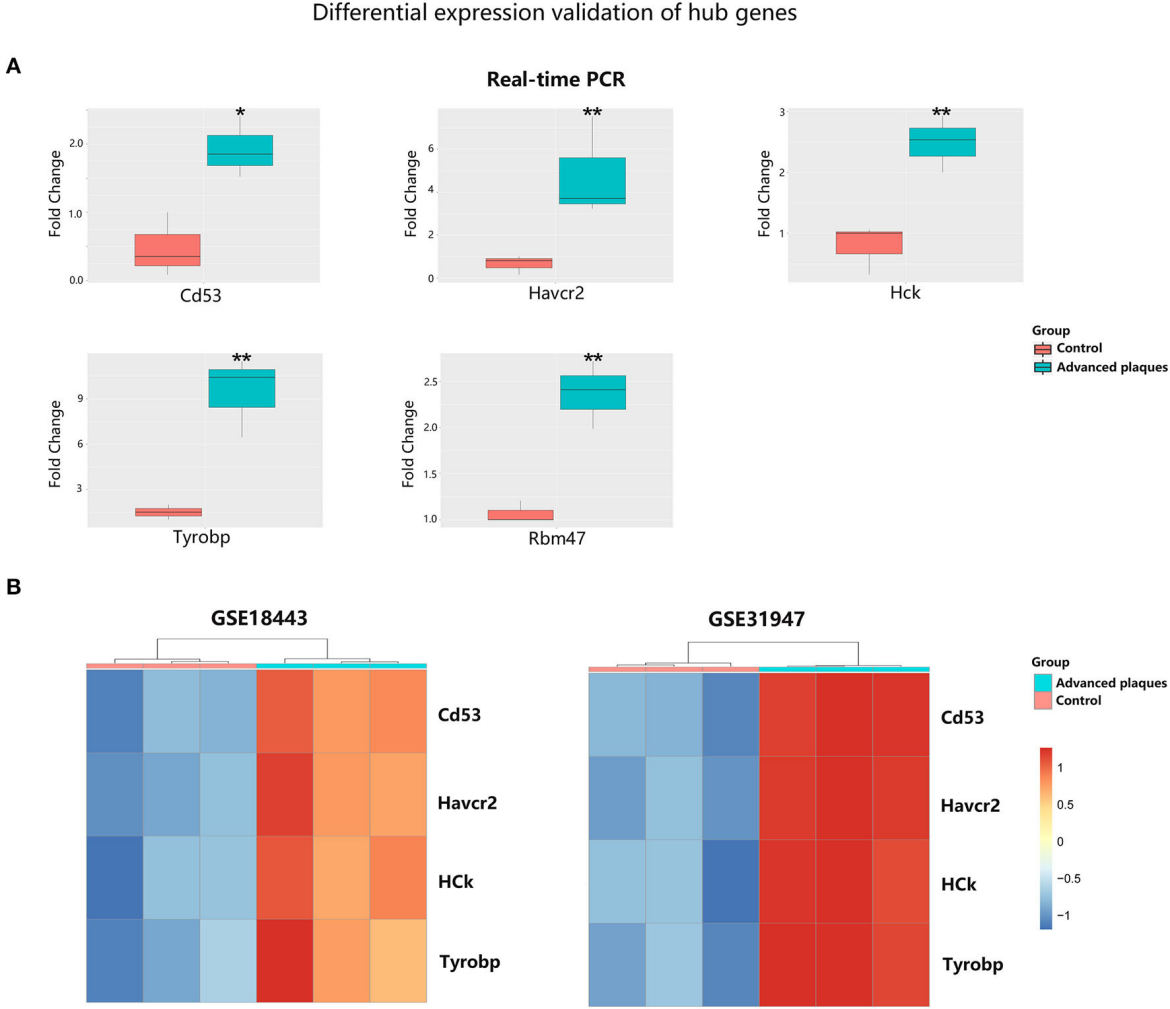
Validation of hub gene expression in experimental atherosclerosis models. **(A)** Quantitative real-time PCR of ApoE^−/−^ mice aortic tissues. The data are shown as the relative fold change in expression. **(B)** Heatmap of differentially expressed hub genes in dataset GSE18443 and GSE31947. *p < 0.05, **p < 0.01.

To validate the expression of these five hub genes in advanced plaque formation, we constructed a mouse model by feeding ApoE^−/−^ mice with high fat-diet for 16 weeks. As shown in Figure 7A, mRNA levels of Rbm47, Havcr2, Hck, Cd53, and Tyrobp were significantly upregulated in advanced plaques group, compared to control group (*P* < 0.05). We further validated the expression of these genes in three other datasets. As shown in Figure 7B, with the threshold of *P* < 0.05, Havcr2, Hck, Cd53, and Tyrobp were significantly upregulated in advanced plaques group in both dataset GSE18443 and GSE31947 (the platform GPL3677 did not detect Rbm47 expression). Besides, CD53, HCK, TYROBP, and RBM47 were significantly upregulated in patients with coronary atherosclerotic disease in dataset GSE12288 (Supplementary Figure 3, the platform GPL96 did not detect HAVCR2 expression).

### ROC Validation of Hub Genes in Coronary Atherosclerotic Disease

**Figure 8:**
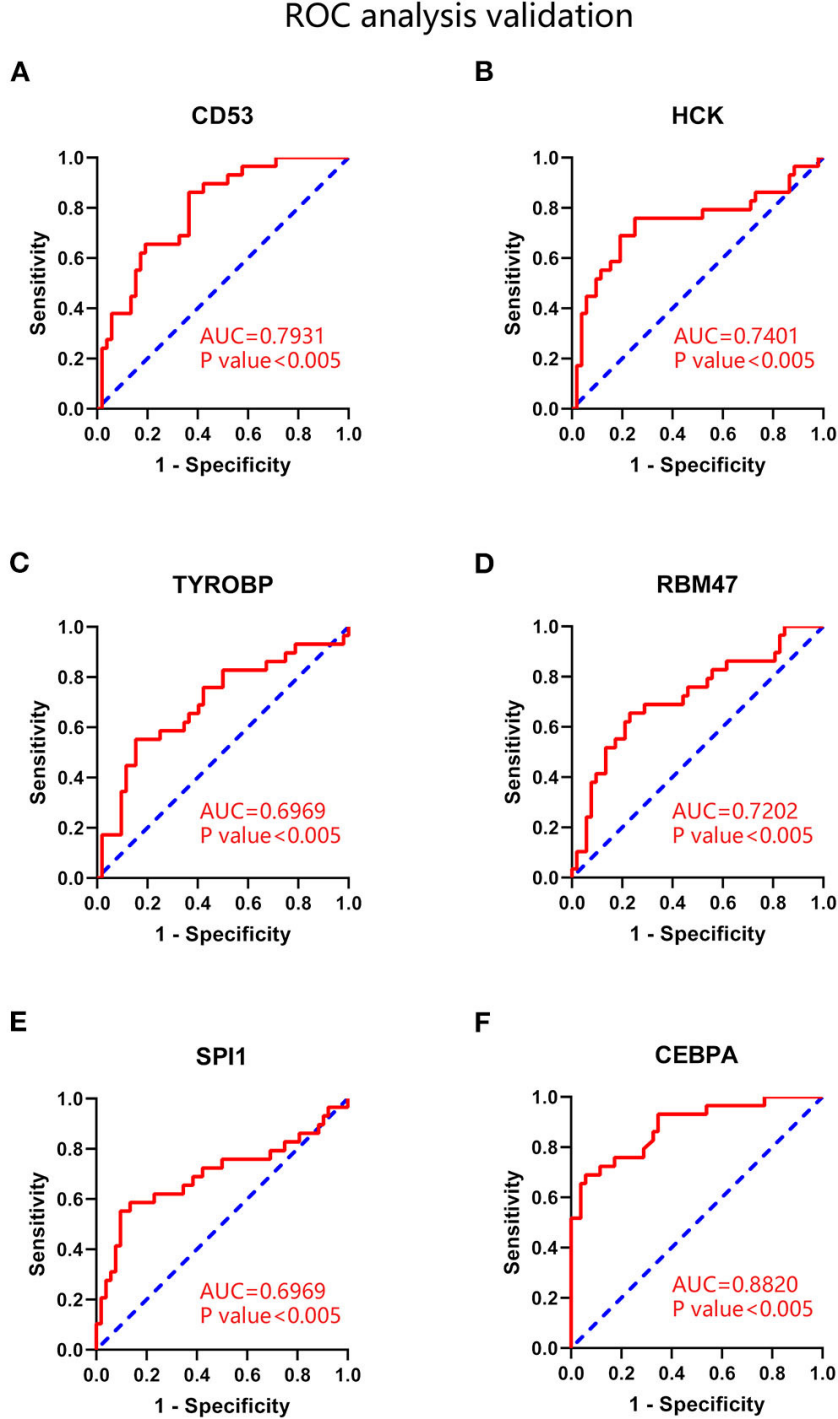
ROC analysis of hub genes and key transcription factors in coronary atherosclerotic disease. **(A)** CD53. **(B)** HCK. **(C)** TYROBP. **(D)** RBM47. **(E)** SPI1. **(F)** CEBPA. AUC statistics is to evaluate the capacity of distinguishing CAD and non-CAD group.

ROC analysis was performed to evaluate the capacity of these hub genes to distinguish samples of CAD group from those of non-CAD group. As shown in Figures 8A–D, the expression of CD53, HCK, RBM47 represented high efficiency in distinguishing capacity of CAD (AUC >0.7). The expression of TYROBP showed modest efficiency in distinguishing capacity of CAD (AUC = 0.6969). The platform GPL96 did not detect HAVCR2 expression.

### GSEA of Hub Gene Function in Advanced Atherosclerosis

To further explore the potential functions of CD53, HCK, RBM47, TYROBP, and HAVCR2 in advanced atherosclerosis, GSEA was performed on these hub genes, respectively. As shown in Supplementary Figure 4, genes in high expression groups of CD53, HCK, RBM47, TYROBP, and HAVCR2 were all enriched in lysosome; genes in high expression groups of HCK and RBM47 were both enriched in complement and coagulation cascades and sphingolipid metabolism; genes in high expression group of CD53 were enriched in Toll like receptor signaling and vibrio cholerae infection; genes in high expression group of HAVCR2 were enriched in T cell receptor signaling and B cell receptor signaling genes in high expression group of TYROBP were enriched in NOD like receptor signaling and natural killer cell mediated cytotoxicity. Collectively, these results suggested that these hub genes regulated immune cell function-related mechanisms in advanced atherosclerosis.

### Identifying Key Transcriptional Regulatory Networks in Advanced Plaque-Related Modules

To identify key transcriptional regulatory networks that control these hub genes, TRRUST database was used. As shown in Supplementary Table 3, there were 12 transcription factor-mediated regulatory networks were significantly enriched in MElightgreen, and 24 transcription factor-mediated regulatory networks were significantly enriched in MEbrown. SPI1, RELA, NFKB1, ETS1, SP1, ERG, STAT1, STAT3, TRERF1, ETS2, and CEBPA mediated regulatory networks were enriched in both MElightgreen and MEbrown.

Then differential expression analysis and ROC analysis were applied to validate these transcription factors. As shown in Supplementary Table 4, only SPI1 and CEBPA were significantly upregulated in advanced plaques (*P* < 0.05). Subsequent ROC analysis demonstrated that the expression of CEBPA showed high efficiency in distinguishing capacity of CAD (AUC = 0.8820). The expression of SPI1 showed modest efficiency in distinguishing capacity of CAD (AUC = 0.6969), as shown in Figures 8E,F. These results indicated that SPI1 and CEBPA played key transcriptional regulatory roles in advanced atherosclerosis.

### PPI Network Construction and Analysis

To better understand the protein-protein interactions of hub genes in advanced plaques based on prior knowledge, PPI network was constructed. As shown in Supplementary Tables 5, 6, there were 2,972 interactions identified in the PPI network of MElightgreen, and 2,240 interactions identified in the PPI network of MEbrown based on previous evidence. By calculating node degree, the 10 genes with the highest degree in MElightgreen and MEbrown were identified separately (Supplementary Table 7). Importantly, PTPRC, TYROBP, ITGB2, CD86, PLEK, LCP2, TLR2, TLR8, and CSF1R were the common high-degree genes.

## Discussion

In this study, two advanced atherosclerotic plaque-related microarray datasets were analyzed by WGCNA to identify gene sets (modules) and their hub genes that were most related to advanced plaques. Functional enrichment analysis showed that advanced plaques-related modules were enriched in immune-related mechanism. The common hub genes of advanced plaque-related modules between two datasets were further validated by quantitative real-time PCR in advanced atherosclerotic tissue and by other datasets. Besides, these hub genes were also validated for distinguishing capacity of CAD through ROC analysis and for potential functions in advanced atherosclerosis through GSEA.

Weighted gene co-expression network analysis (WGCNA) was the main analytical method in this study. It can be used to identify gene sets (modules) of highly co-expressed genes, to summarize such gene sets using module eigengene (ME) values or intramodular hub genes, to correlate modules with one another and with clinical traits of samples through eigengene network methodology, and to calculate module membership (MM) values of genes (Zhang and Horvath, 2005). In addition, WGCNA is based on the idea of correlation networks rather than individual genes which can effectively identify candidate biomarkers or therapeutic targets. This analytical method has been successfully applied in diverse biomedical fields, like cancer, model organism genetics and analysis of neuroimaging data (Carlson et al., 2006; Ghazalpour et al., 2006; Horvath et al., 2006; Weston et al., 2008). In the present study, we identified advanced plaque-related gene co-expression networks through WGCNA and found they were associated with immune-related pathways, indicating the central role of immune mechanisms in advanced plaque formation. In recent decades, immune and inflammatory mechanisms have become increasingly important in experimental atherosclerosis studies (Minelli et al., 2020). Many initial factors such as hemodynamic disorder, endothelial dysfunction and subendothelial buildup of cholesterol-carrying LDL trigger innate and adaptive immune responses, which affect plaque progression through a complex interaction network, balancing pro-atherogenic inflammatory and atheroprotective anti-inflammatory responses (Libby and Hansson, 2015). Additionally, interleukin 1β-targeted monoclonal antibody canakinumab significantly lowered the rate of recurrent cardiovascular events in previous myocardial infarction patients, indicating the potential of immunotherapy in atherosclerosis progression (Ridker et al., 2011). Howerver, rather than broad-spectrum anti-inflammatory therapy, investigation of specific inhibition of key targets is warranted (Minelli et al., 2020).

In this study, RBM47, HCK, CD53, TYROBP, and HAVCR2 were identified as hub genes in plaque progression. RNA-Binding-Motif-protein-47 (RBM47), a component of the editosome, has been implicated in the editing of Apob mRNA *in vivo* and *in vitro*, which affects the production of Apob functional proteins APOB100 and APOB48 (Fossat et al., 2014). ApoB is a cholesterol-carrying component of LDL with a well-known atherogenic effect on artery subendothelial lipid retention and accumulation (Skalen et al., 2002). But it remains to be clarified that RBM47 functions as an RNA editing factor for ApoB in immune cells and atherosclerotic plaques tissue. HCK is a member of the Src family of tyrosine kinases, which transmits membrane receptor signals and plays an important role in survival, proliferation migration and phagocytosis of immune cells (Wang et al., 2020). HCK has also been reported to participate in leukocyte adhesion and metastasis, which may promote atherosclerotic plaque formation (Medina et al., 2015). However, the specific mechanism of HCK in atherosclerosis development still need further elaboration. CD53 is a member of the tetraspanin family and mediates signal transduction functions (Yeung et al., 2020). It has been suggested that CD53 regulated the growth of T cells and natural killer cells and has been extensively studied in infectious diseases, autoimmune diseases, and immunodeficiency diseases (Kim et al., 2004; Tohami et al., 2004; Pedersen-Lane et al., 2007). Interestingly, CD53 has been shown to curb inflammatory cytokines secretion and pathway activation of THP-1 cells, which is the human monocyte and widely used to construct atherosclerotic models *in vitro* (Lee et al., 2013). Hepatitis A virus cellular receptor 2 (HAVCR2, also known as Tim-3), a cell surface receptor, was widely believed to play an inhibitory role by associating with some molecules like ZAP70, LCP2, LCK, and FYN and blocking phosphorylation of key components of signaling pathways (Monney et al., 2002). It was reported that HAVCR2 could regulate macrophage activation and inhibit T-helper type 1 lymphocyte-mediated auto- and alloimmune responses and promote immunological tolerance (Monney et al., 2002; Sánchez-Fueyo et al., 2003). Amanda C Foks, et al. showed that anti-Tim-3 antibody treatment promoted plaque formation in atherosclerosis model, increased lesional percentages of macrophages and CD4(+) T cells and enhanced their activation (Foks et al., 2013). However, the immunosuppressive mechanisms of HAVCR2 against atherosclerosis remains to be elucidated. TYROBP, also known as DNAX-activating protein of 12 kDa (DAP12), encodes immune-related signal transduction adaptin on the membrane and works through an immunoreceptor tyrosine-based activation motif (Kobayashi et al., 2015). DAP12 was widely involved in the proliferation, survival, differentiation, and polarization of immune cells, especially monocyte-macrophage system (Otero et al., 2009; Kobayashi et al., 2015). Interestingly, TYROBP was also identified as the high-degree gene in the PPI network of advanced plaques, indicating its core role in advanced atherosclerosis.

## Conclusions

In summary, our present study has identified RBM47, HCK, CD53, TYROBP, and HAVCR2 as hub genes in advanced plaque progression by weighted gene co-expression network analysis. They were validated to be upregulated during advanced plaque progression, to well distinguish CAD patients from non-CAD people and to regulate immune cell function-related mechanisms in advanced atherosclerosis. Our results provide noval molecuar targets for future research. These hub genes may also serve as promising therapeutic intervention targets to inhibit advanced plaque progression.

## Data Availability Statement

The original contributions presented in the study are included in the article/Supplementary Materials, further inquiries can be directed to the corresponding author/s.

## Ethics Statement

The animal study was reviewed and approved by Institutional Animal Care and Use Committee, SYSU.

## Author Contributions

ZL and JW directed the project. HZ and SW designed experiments. CL performed experiments and drafted the manuscript. YC and ZC performed data analysis. All authors reviewed the manuscript, read, and approved the final manuscript.

## Conflict of Interest

The authors declare that the research was conducted in the absence of any commercial or financial relationships that could be construed as a potential conflict of interest.
