# Supplementary figures and images for: Identifying RBM47, HCK, CD53, TYROBP, and HAVCR2 as Hub Genes in Advanced Atherosclerotic Plaques by Network-Based Analysis and Validation
Source: Front Genet. 2021 Jan 15;11:602908. doi: 10.3389/fgene.2020.602908 (PMC7844323; doi:10.3389/fgene.2020.602908)

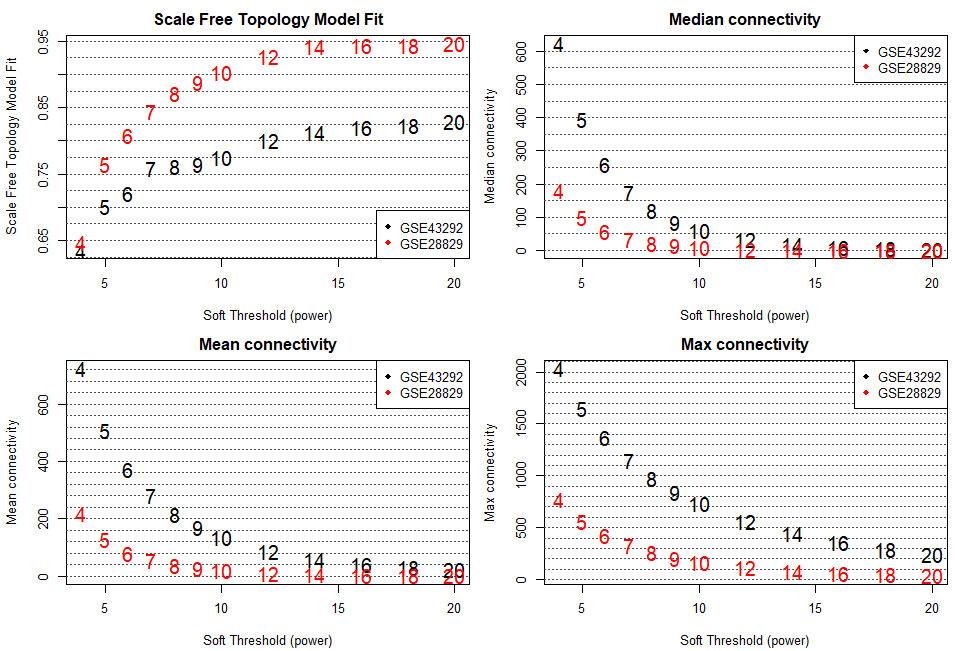

Supplement: Supplementary file 8 [file Image_1.TIFF]

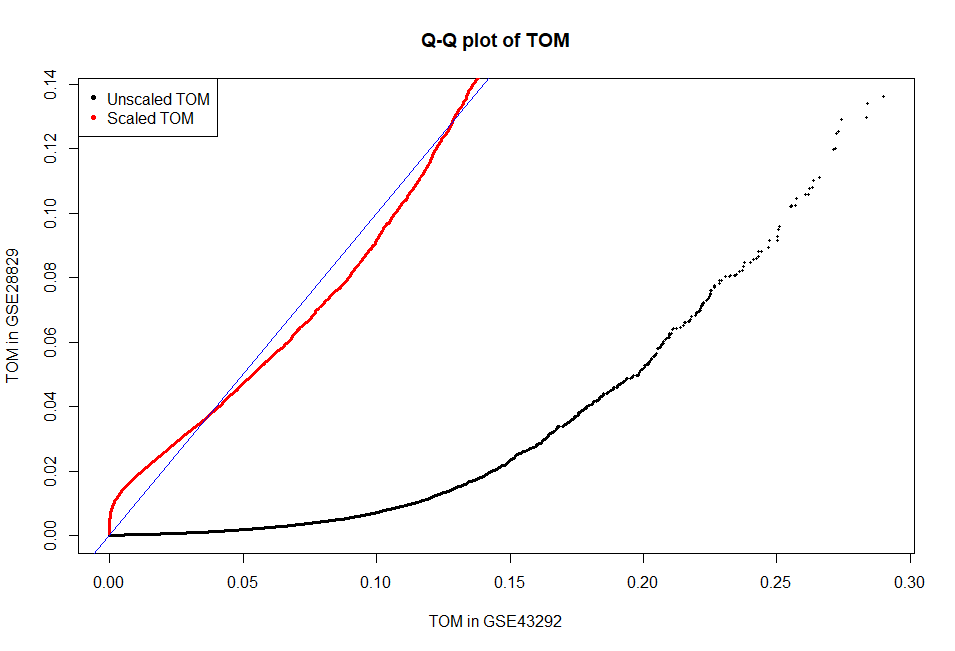

Supplement: Supplementary file 9 [file Image_2.TIFF]

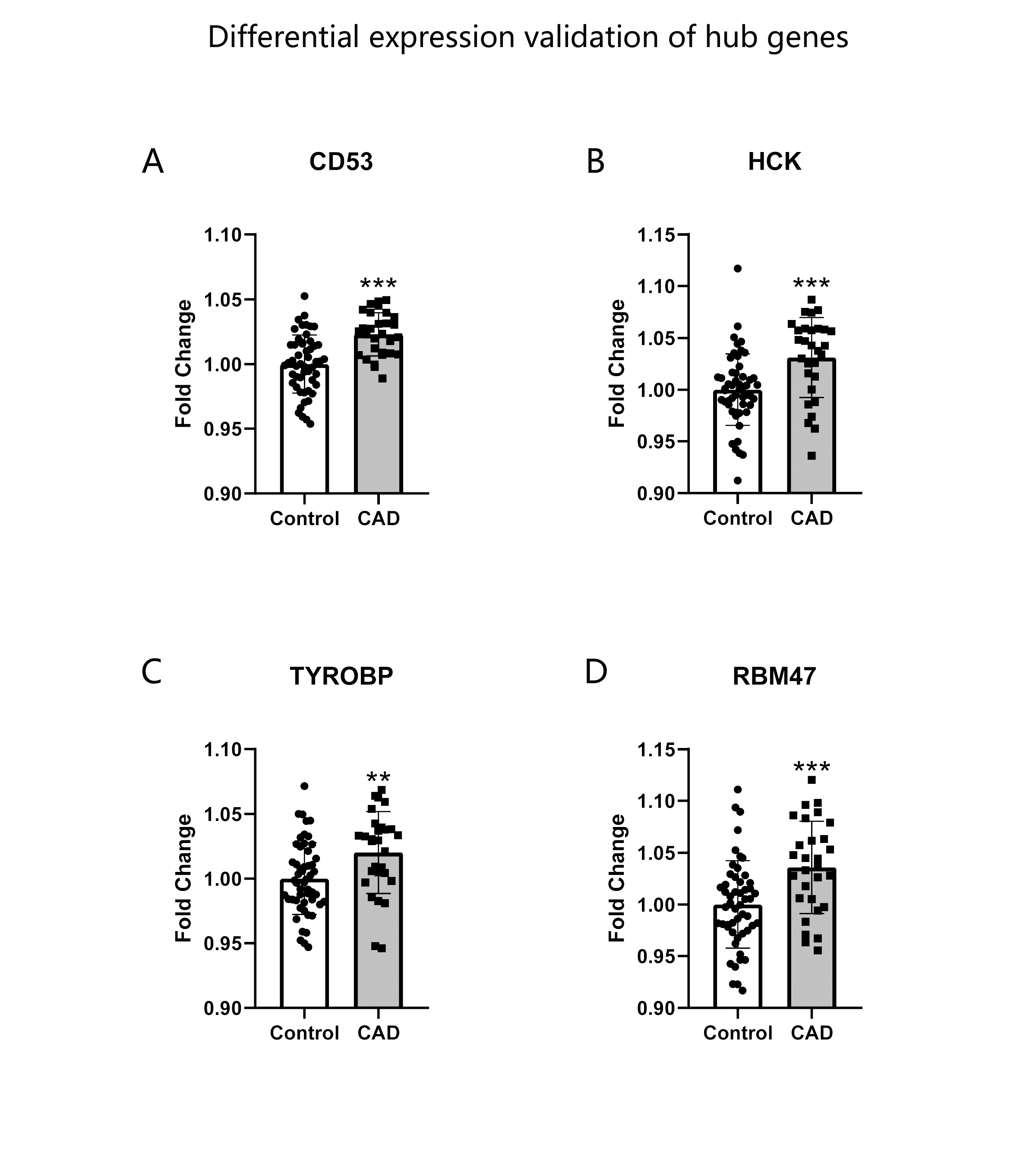

Supplement: Supplementary file 10 [file Image_3.TIFF]

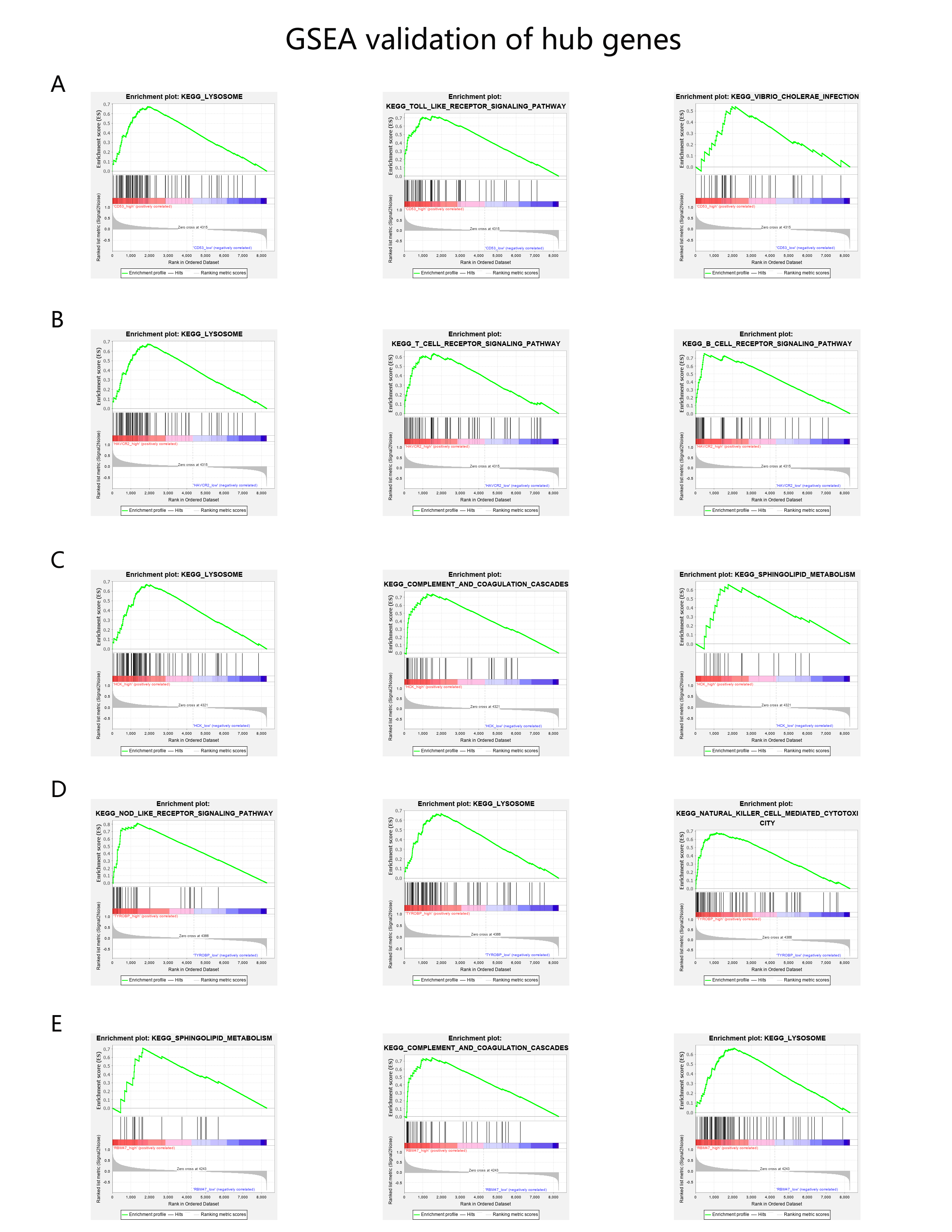

Supplement: Supplementary file 11 [file Image_4.TIF]
